# Post-induction serum vedolizumab levels are not associated with better maintenance outcomes in patients with Crohn’s disease

**DOI:** 10.1055/a-2744-5136

**Published:** 2026-01-12

**Authors:** Jagoda Pokryszka, Christian Primas, Michael Stadlmann, Nathalie Gerold, Cornelia Lichtenberger, Sieglinde Reinisch, Gottfried Novacek, Walter Reinisch

**Affiliations:** 1 27271 Department of Internal Medicine III, Division of Gastroenterology and Hepatology, Medical University of Vienna, Wien, Austria; 2 Magen-Darm-Gesundheitszentrum, Mödling, Austria

**Keywords:** drug level monitoring, vedolizumab, exposure-outcome relation, Crohn’s disease, trough level, therapeutisches Drug Monitoring, Vedolizumab, chronisch entzündliche Darmerkrankung, Morbus Crohn, Darm

## Abstract

**Aim:**

Results on exposure-efficacy relationships for vedolizumab in patients with Crohn’s disease are contentious. Our study aimed at exploring the relationship between vedolizumab serum concentrations measured during induction and maintenance and treatment outcomes in Crohn’s disease patients in a real-world setting.

**Methods:**

Crohn’s disease patients treated with vedolizumab between January 2014 and July 2022 at our tertiary care centre were included. Serum vedolizumab concentrations were measured on at least one of the following time points: week 2, 6, 12, 26, and/or 52. Treatment efficacy was evaluated at weeks 12, 26 and 52 as clinical remission based on patients reported outcomes, faecal calprotectin and C-reactive protein remission.

**Results:**

In total, 58 patients (55.2% females) were included. At baseline, active disease, defined either by increased faecal calprotectin, C-reactive protein or patients reported outcomes, was observed in 53 patients (91.4%). Week 12 vedolizumab serum levels were higher in clinical remitters versus non-remitters at weeks 12 and 52 (p <0.05). However, in adjusted multivariate analyses no association between vedolizumab levels and therapy outcomes was observed.

**Conclusion:**

In our retrospective study, vedolizumab serum concentrations measured during induction treatment were not associated with clinical and feacal calprotecin remission at weeks 12, 26 and 52.

## Zusammenfassung

**Hintergrund:**

Die großen randomisierten Studien haben einen Zusammenhang zwischen Vedolizumab-Talspiegeln und Ansprechen auf diese Therapie bei Patienten mit Morbus Crohn gezeigt. Die sog. “real-world”-Studien haben allerdings ein heterogenes Bild ergeben. Das Ziel dieses Projekts war daher, diese Assoziation genauer zu untersuchen.

**Methodik:**

Alle Morbus-Crohn-Patienten, die zwischen 01/2014 und 07/2022 an unserem tertiären Zentrum mit Vedolizumab behandelt wurden und von denen wir über min. eine Vedolizumab-Talspiegelmessung verfügten (Woche 2, 6, 12, 26 oder 52), wurden inkludiert. Das Ansprechen wurde zur Woche 12, 26 und 52 anhand der klinischen Faktoren (PROs) oder biochemischen Marker (fäkales Calprotectin (fCP); C-reaktives-Protein (CRP)) evaluiert. Die Assoziation zwischen den Talspiegeln und der Remission wurde mittels logistischer Regression berechnet.

**Ergebnisse:**

Insgesamt wurden 58 Patienten (davon 55,2% Frauen) inkludiert. Zu Beginn der Therapie hatten 53 Patienten (91,4%) eine aktive Erkrankung, die durch erhöhtes fCP, CRP oder PROs definiert wurde. Die Vedolizumab-Talspiegel zur Woche 12 (nach der Induktionsphase) waren höher bei denjenigen, die zur Woche 12 und 52 eine klinische Remission erreicht haben (p<0,05). In einer adjustierten multivariablen Analyse wurde allerdings der Zusammenhang zwischen diesen Talspiegeln und dem Ansprechen nicht bewiesen.

**Zusammenfassung:**

In unserer retrospektiven Studie konnte keine Assoziation zwischen den Vedolizumab-Talspiegeln und dem klinischen oder biochemischen Ansprechen bei Patienten mit Morbus Crohn festgestellt werden.

## Abbreviations

BL: baseline
CD: Crohn’s disease
CRP: C-reactive protein
fCP: fecal calprotectin
GI: gastrointestinal
IBD: inflammatory bowel disease
IQR: interquartile range
MAdCAM-1: mucosal addressin cell adhesion molecule-1
PK: pharmacokinetics
PRO: patients reported outcomes
RWE: real-world evidence
TDM: therapeutic drug monitoring
TNFα: tumor necrosis factor α
UC: ulcerative colitis
VDZ: vedolizumab

## Introduction

Biologics pose a mainstay in the treatment of patients with inflammatory bowel diseases (IBD), with the longest experience for those blocking tumor necrosis factor α (TNFα). In clinical trials the dose selection process of biologics includes pharmacokinetic/pharmacodynamic modelling and exposure response relationship assessments. Nonetheless, the introduction of therapeutic drug monitoring (TDM) of anti-TNF agents took up to a decade from approval to gain traction as a potential tool to optimize treatment outcome in patients with IBD. Whereas initial pharmacokinetic (PK) results from controlled IBD studies on infliximab were contentiously discussed 1 2 3 , the meanwhile accumulated wealth of evidence on anti-TNF agents suggests that proactive TDM is associated with significant benefit in reducing treatment failure, including hospitalizations 4 .

Vedolizumab (VDZ) is approved for the treatment of both moderately to severely active ulcerative colitis (UC) and Crohn’s disease (CD). As an integrin antagonist, VDZ blocks the interaction of α4β7 on the surface of mononuclear cells, including gut-homing T cells 5 , and the mucosal addressin cell adhesion molecule-1 (MAdCAM-1) expressed by the endothelial cells of the gastrointestinal (GI) tract. The mechanism of action of VDZ, however, appears more complex than only impeding pro-inflammatory cell homing into the mucosa, substantiated by an almost universal α4β7 receptor occupancy on both peripheral and lamina propria T cells despite low drug serum concentrations 6 . Vedolizumab clearance does not depend on the type of IBD as a combined analysis of pivotal clinical trial data suggested 5 . Nonetheless, stronger VDZ exposure response relationships during induction treatment have been reported for UC than CD 7 and a predictive value of post-induction week 6 VDZ serum concentrations for post-maintenance clinical remission has been suggested 8 . In CD higher VDZ concentrations as early as at week 2 were reported to predict C-reactive-protein-based biologic remission at week 6 9 . However, a TDM-based dose or interval adjustment did not result in higher clinical remission rates in UC patients non-responding at week 6 10 , suggesting a complex exposure-outcome relationship that is even less interrogated in patients with CD.

At our center, measurement of VDZ serum concentrations is part of the routine management. Here, we explored from our real-world CD cohort relationships between VDZ serum concentrations measured at multiple time points during induction and maintenance treatment and clinical and/or biomarker defined endpoints.

## Methods

The study included consecutive patients with CD in their first year of VDZ treatment at the Department of Gastroenterology and Hepatology, Medical University of Vienna between January 2014 and July 2022 as part of a program investigating the prescription practice and treatment outcomes of biologics in CD patients treated at our center. Here, we aimed to assess drug exposure-outcome relationships during the first year of the treatment with VDZ.

Only individuals for whom data at baseline and a VDZ serum concentration on at least one of the following time points, week 2, 6, 12, 26, and/or 52 were available, were considered for study eligibility. Demographic data, disease characteristics and laboratory results were obtained from electronic hospital records and collected at baseline (BL), which was defined within 2 months prior to therapy start. Data for outcome measures was withdrawn from the same source at week 12 (+/– 2 weeks, later called “post-induction”), week 26 (+/– 4 weeks) and week 52 (+/– 6 weeks). Week 26 and week 52 are referred to as the maintenance endpoints. The following separate outcome measures were evaluated and defined as remission state:

- Clinical remission: as assessed by patient reported outcomes (PROs) and defined as stool frequency ≤ 3/day and abdominal pain score ≤ 1
- Calprotectin remission: fecal calprotectin (fCP) < 150 µg/g
- C-reactive protein (CRP) remission: reduction of min. 50% compared to baseline and normalization < 0.5 mg/dl.

Each of those outcomes were only measured in patients who at BL were clinically, fCP or CRP active, respectively. Clinical activity was defined as stool frequency > 3 bowel movements/day or abdominal pain score > 1, calprotectin activity as fCP ≥ 150 µg/g and CRP activity as CRP ≥ 0.5 mg/dl. If a patient did not achieve an individual remission criterion at the end of the induction, primary treatment failure for that criterion was stated. Secondary treatment failure applied to initial remitters at any visit who lost that status subsequently. Abdominal pain score was defined as follows: 0 – no pain, 1 – mild pain, 2 – moderate pain, 3 – very strong pain.

Vedolizumab serum drug levels were measured with IDKmonitor Vedolizumab drug level ELISA kit (Immundiagnostik AG, Germany). For measurement of fCP values BUHLMANN fCAL ELISA kit (Buhlmann Diagnostics Corp, Amherst, NH) was used according to the manufacturer’s instructions. As patients are always instructed to have their blood sampling performed at the end of the dose interval, at longest 2 days prior administration, we may interpret data from weeks 26 (+/– 4 weeks) and 52 (+/– 6 weeks) as trough concentrations. Those from week 12 (+/– 2 weeks) are trough post-induction values.

Dichotomous variables are presented as percentages, the continuous variables as medians with interquartile ranges (IQR). Shapiro-Wilk-test was applied to check whether continuous variables follow normal distribution. No data imputation was performed. For group comparisons two-sided Mann-Whitney-U-test was used (in case of multiple testing, correction by Benjamin-Hochberg was performed). For testing associations between VDZ serum concentrations and outcomes, simple logistic regression was used. As potential confounding variables based on the literature search, we included: sex, as well as age, smoking status, disease duration, concomitant corticosteroids and serum CRP levels at baseline. Co-variates meeting the pre-defined inclusion criteria by Bursac et al (inclusion criterium = 0.25, retention criterium = 0.1), entered the multivariate regression model 11 . No power analysis for sample size estimation was conducted. The significance level was set at α = 0.05. All statistical analyses were carried out in R v.4.2.1. (R Core Team, Vienna, Austria).

The project was approved by the Ethics Committee of the Medical University of Vienna (1904/2019). The study was performed in accordance with Declaration of Helsinki 1964. The patients underwent only routine examinations and no additional interventions.

## Results

### Study population

In total, 58 patients (females n = 32; 55.2%) were included. The median age at BL was 43.2 (IQR: 28.1) years. Most of patients had either colonic (n = 28, 48.3%) or ileocolonic CD (n = 19, 32.8%). Additional manifestations of the upper gastrointestinal tract were present in 13.8% patients (n = 8). The majority of patients were previously exposed to TNFα-inhibitors (n = 42, 72.4%). At baseline five patients (8.6%) concomitantly received corticosteroids and 10 (17.2%) patients immunosuppressants including azathioprine, 6-mercaptopurine and methotrexate. Demographic data and disease characteristics are summarized in Table 1 .

**Table:** **Table 1** Baseline demographics and disease characteristics.

Characteristic	All patients
	N = 58
**Female sex – no. (%)**	32 (55.2)
**Age at baseline [years] – median (IQR)**	43.2 (28.1)
**Disease duration [years] – median (IQR)**	12.1 (12.8)
**Location of disease- no. (%)**	
**Ileal only**	11 (19.0)
**Colonic only**	28 (48.3)
**Ileocolonic**	19 (32.7)
**Upper gastrointestinal disease**	8 (13.8)
**Behaviour – no. (%)**	
**Non-stricturing, non-penetrating**	37 (63.8)
**Stricturing**	15 (25.9)
**Penetrating**	6 (10.3)
**Perianal disease – no. (%)**	6 (10.3)
**Extraintestinal manifestations – no. (%)**	25 (43.1)
**Previous intestinal surgeries – no. (%)**	19 (32.8)
**Prior medications – no. (%)**	
**Azathioprine**	31 (53.4)
**6-Mercaptopurine**	7 (12.1)
**Methotrexate**	7 (12.1)
**Adalimumab**	32 (55.2)
**Infliximab**	24 (41.4)
**Corticosteroids at baseline – no. (%)**	5 (8.6)
**Immunosuppressants at baseline – no. (%)**	10 (17.2)
**Smoking at baseline – no. (%)**	
**Active smokers**	10 (17.2)
**Ex-smokers**	15 (25.9)
**Non-smokers**	30 (51.7)
**Unknown**	3 (5.2)
**Abdominal pain score – median (IQR)**	1.0 (2.0)
**Stool frequency – median (IQR)**	4.0 (4.0)
**fCP [µg/g] – median (IQR)**	884 (1585.8)
**CRP [mg/dl] – median (IQR)**	0.37 (1.51)
Categorical variables are presented as absolute numbers and proportions of total, continuous variables as medians with interquartile ranges (IQR). For abdominal pain the following score was used: 0 – no pain, 1 – light pain, 2 – moderate pain, 3 – very strong pain. CRP – C-reactive protein; fCP – fecal calprotectin	

Within the first year of treatment, 15.5% of patients (9/58) who started on intravenous VDZ switched to the subcutaneous formulation. Taking into account that subcutaneous vedolizumab was approved by the European Medicines Agency in May 2020 and reimbursed by Austrian public health insurance in October 2020 it could only be administered to 13 patients during that period. All patients received 3 vedolizumab infusions during the induction period (BL, week 2 and week 6).

At the end of induction, 57 out of 58 patients (98.3%) stayed on vedolizumab treatment (one patient was lost to follow-up) with corresponding number at week 26 and week 52 of 53/58 (91.4%) and 48/58 (82.8%) individuals, respectively. Reason for discontinuation at week 26 were primary treatment failure (3/5, 60%), secondary treatment failure (1/5, 20%), and death unrelated to IBD or IBD treatment (terminal renal and heart insufficiency) (1/5, 20%) and at week 52 were secondary treatment-failure (4/5, 80%) and non-compliance (1/5, 20%).

Vedolizumab was generally well tolerated with adverse events reported in total by 14/58 subjects (24.1%), most frequently as dermatological manifestations (6/58, 10.3%, 2 cases of exanthemas, 2 cases of pruritus, one case of herpes zoster, one case of folliculitis) and infections (3/58, 5.2%).

### Treatment outcomes

**Fig. 1:**
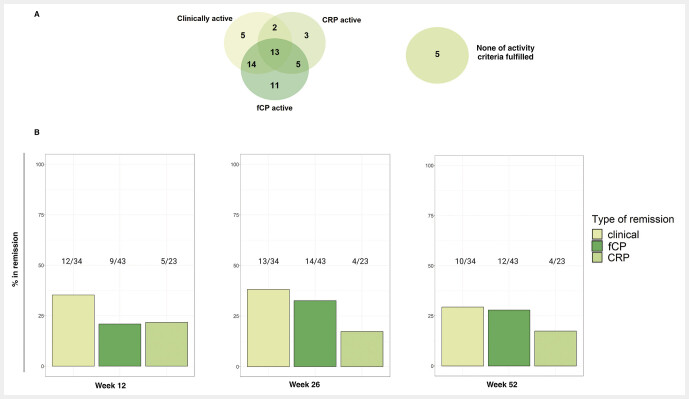
Disease activity at baseline and treatment outcomes to vedolizumab in patients with Crohn’s disease at week 12, 26 and 52. **A** . Venn diagram displaying distribution of various types of disease activity at baseline. The definitions of baseline activity are provided in the Methods section. **B** . Bar plots show proportion of patients within each active disease category achieving the pre-defined remission criteria at week 12, 26 and 52. Numbers above the bars indicate absolute numbers of patients in remission/active patients at baseline. CRP – C-reactive protein, fCP – fecal calprotectin.

At baseline our post-hoc definition of clinically active CD, fCP active CD and CRP active CD applied to 58.6% (34/58,) 74.1% (43/58) and 39.7% (23/58) of patients, respectively ( Fig. 1 A), leaving 5/58 (8.6%) individuals inactive on all levels of explored disease activity. Notably, those inactive patients were steroid-dependent or intolerant to infliximab with presence of anti-drug antibodies. Only 13 individuals (13/58, 22.4%) fulfilled all three definitions of disease activity explaining why analyses on subgroups with combined efficacy outcomes were not pursued.

Treatment outcomes at weeks 12, 26 and 52 were assessed only within each active disease subgroup ( Fig. 1 B). Among all 34 patients who were clinically active at BL, 35.3% (12/34), 38.2% (13/34) and 29.4% (10/34) achieved clinical remission at week 12, 26 and 52, respectively. The corresponding proportions of patients in fCP remission were lower: 20.9% (9/43), 32.6% (14/43) and 27.9% (12/43). Among the 23 baseline CRP-active subjects 21.7% (5/23) met the corresponding remission criterion at week 12. At weeks 26 and 52 these percentages were each 17.4% (4/23).

### Association between vedolizumab serum levels and treatment outcomes

Population-based median VDZ levels at week 2 were 22.2 µg/ml (IQR: 15.8 µg/ml; n = 43) and at week 6 19.8µg/ml (IQR: 22.2 µg/ml; n = 47; Table 2 ). Post-induction (W12) median drug levels were 15.9 µg/ml (IQR: 9.8 µg/ml; n = 33) and those at week 26, 9.1 µg/ml (IQR: 8.8 µg/ml; n = 27) and at week 52, 11.4 µg/ml (IQR: 18.7 µg/ml; n = 22).

**Table:** **Table 2** Summary of main laboratory parameters.

	Median	IQR	Available values (total at baseline; n = 58)
**Timepoint**			
**Week 2**			
**CRP [mg/dl]**	0.32	0.98	52
**fCP [µg/g]**	439.5	1258.5	50
**VDZ level [µg/ml]**	22.2	15.8	43
**Week 6**			
**CRP [mg/dl]**	0.27	0.79	51
**fCP [µg/g]**	243	871.5	47
**VDZ level [µg/ml]**	19.8	22.2	47
**Week 12**			
**CRP [mg/dl]**	0.51	0.88	45
**fCP [µg/g]**	309	1143.8	40
**VDZ level [µg/ml]**	15.9	9.8	33
**Week 26**			
**CRP [mg/dl]**	0.25	0.78	36
**fCP [µg/g]**	184	1160	39
**VDZ level [µg/ml]**	9.1	8.8	27
**Week 52**			
**CRP [mg/dl]**	0.2	0.37	34
**fCP [µg/g]**	165.5	357.5	34
**VDZ level [µg/ml]**	11.4	18.7	22
The table shows median and interquartile ranges (IQR) as well as the number of available values for C-reactive protein (CRP), fecal calprotectin (fCP) and population vedolizumab serum levels (VDZ level for all patients at weeks 2, 6, 12 (+/– 2 weeks), 26 (+/– 4 weeks), 52 (+/– 6 weeks).			

**Fig. 2:**
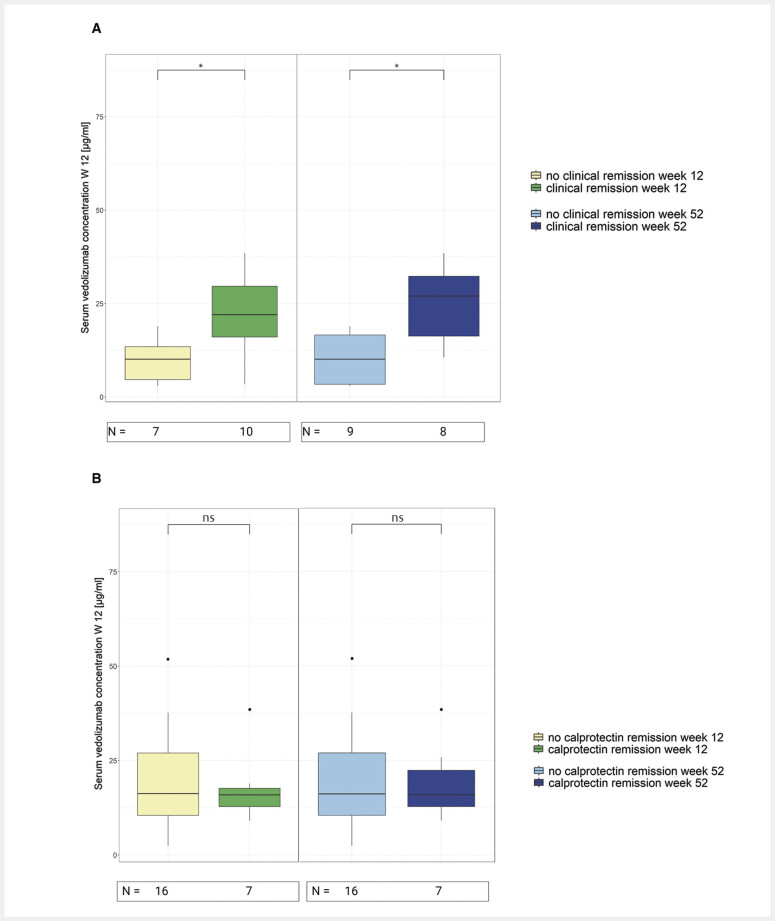
Comparison of vedolizumab levels in serum between remitters and non-remitters. **A** Vedolizumab concentration measured at week 12 after baseline in clinically active patients compared between those who achieved clinical remission at week 12 and those who did not (left side) and those who achieved clinical remission at week 52 and non-remitters. **B** Vedolizumab concentration measured at week 12 after baseline in calprotectin active patients compared between those who achieved calprotectin remission at week 12 and those who did not (left side) as well as those who achieved calprotectin remission at week 52 and non-remitters (right side). The comparisons were performed with Mann-Whitney-U-tests corrected for multiple testing with Benjamin-Hochberg method. Numbers under the boxplots indicate the number of patients with available vedolizumab serum concentration in each subgroup. ns – not significant, * – p < 0.05, fCP – faecal calprotectin.

We compared VDZ serum levels during induction phase (weeks 2 and 6) and from post-induction (week 12) between patients with and without remission for each active disease stratum at week 12, 26 and 52. Notably, there were only few statistically significant differences between the groups ( Fig. 2 ). At week 2, both future (W12, W26, W52) remitters and non-remitters (all remission types) presented with similar VDZ serum concentrations. Week 6 drug levels were higher for future fCP remitters than non-remitters at W26 (p = 0.03) and W52 (p = 0.03). Post-induction drug levels were also significantly higher in clinical remitters at week 12 (p = 0.03) and week 52 (p = 0.01).

In univariate analyses VDZ serum levels at week 12 were significantly associated with clinical remission at week 52 (p = 0.04, OR = 1.27, 95% CI 1.07 – 1.79; Table 3 ). Likewise, an association between week 6 VDZ serum levels and fCP remission at W26 as well as W52 was observed ( Table 4 ). For the multivariate model additional co-variates were included based on their reported associations with treatment outcomes 12 : sex, age at baseline, smoking, disease duration as well as concomitant steroids and CRP at baseline. As most of the patients had been exposed to tumor necrosis factor α inhibitors, this parameter was not taken into consideration. After adjustment, no association was found between VDZ serum levels and clinical remission at weeks 12 and 52 (p = 0.17, OR = 1.14, 95% CI 0.95 – 1.37; p = 0.15, OR = 1.31, 95% CI 0.91 – 1.89; Table 3 ) or VDZ serum levels measured at week 6 and fCP remission at week 26 (p = 0.21, OR = 1.05, 95% CI 0.98 – 1.13) and week 52 (p = 0.13, OR = 1.06, 95% CI 0.99 – 1.16; Table 4 ).

**Table:** **Table 3** Association between therapy outcomes and parameters.

	Clinical remission W12						Clinical remission W52					
	Univariate Analysis			Multivariate analysis			Univariate Analysis			Multivariate Analysis		
VARIABLES	OR	95% CI	p – value	OR	95% CI	p – value	OR	95% CI	p – value	OR	95% CI	p – value
**age at BL (years)**	1.07	1.02–1.14	0.01	1.04	0.95–1.41	0.21	1.05	1.00–1.10	0.06	1.09	1.00–1.29	0.12
**time to BL (years)**	0.96	0.88–1.05	0.36	NA	NA	NA	0.9	0.77–1.01	0.14	0.88	0.49–1.09	0.38
**Sex**				NA	NA	NA				NA	NA	NA
*male	1	0.26–3.85	1	NA	NA	NA	0.36	0.08–1.75	0.21	NA	NA	NA
*female	Reference			NA	NA	NA	Reference			NA	NA	NA
**Smoking**				NA	NA	NA				NA	NA	NA
*active smoker	0.29	0.04–1.90	0.2	NA	NA	NA	1.12	0.16–7.73	0.91	NA	NA	NA
*ex-smoker	1.45	0.211–9.98	0.7	NA	NA	NA	2.8	0.42–18.69	0.29	NA	NA	NA
*non-smoker	Reference			NA	NA	NA	Reference			NA	NA	NA
**CRP at BL [g/dl]**	0.89	0.71–1.11	0.32	NA	NA	NA	0.92	0.64–1.06	0.48	NA	NA	NA
**steroids at BL**				NA	NA	NA				NA	NA	NA
* yes	0.19	0.03–1.11	0.07	4.96 * 10 ^-9^	0.00–infinity	1	0.22	0.02–2.07	0.19	NA	NA	NA
* no	Reference						Reference			NA	NA	NA
**VDZ level at W12 [µg/ml]**	1.17	1.0–1.38	0.06	1.14	0.95–1.37	0.17	1.27	1.07–1.79	0.04	1.31	0.91–1.89	0.15
The table summarizes the results of univariate and multivariate logistic regression which was used to investigate association between vedolizumab serum levels at week 12 and clinical remission at weeks 12 and 52. Variables that fulfilled pre-specified criteria for incorporation into multivariate modelling. NA –multivariate logistic regression was not performed, BL – baseline, CI – confidence interval, CRP – C-reactive protein, OR – odds ratio, W12 – week 12, W52 – week 52, VDZ – vedolizumab												

**Table:** **Table 4** Association between therapy outcomes and parameters.

	fCP remission W26						fCP remission W52					
	Univariate Analysis			Multivariate Analysis			Univariate Analysis			Multivariate Analysis		
VARIABLES	OR	95% CI	p – value	OR	95% CI	p – value	OR	95% CI	p – value	OR	95% CI	p – value
**age at BL (years)**	1	0.96–1.03	0.85	NA	NA	NA	0.98	0.94–1.02	0.35	NA	NA	NA
**time to BL (years)**	1	0.94–1.06	0.89	NA	NA	NA	0.99	0.92–1.06	0.81	NA	NA	NA
**Sex**												
*male	2.5	0.69–9.77	0.16	2.3	0.39–16.34	0.37	2.5	0.66–10.52	0.18	5.66	0.9–54.22	0.09
*female	Reference			NA	NA	NA	Reference			NA	NA	NA
**Smoking**				NA	NA	NA				NA	NA	NA
*active smoker	1.14	0.13–7.43	0.89	NA	NA	NA	0.38	0.02–2.89	0.41	NA	NA	NA
*ex-smoker	1.09	0.42–8.61	0.39	NA	NA	NA	0.42	0.05–2.14	0.33	NA	NA	NA
*non-smoker	Reference			NA	NA	NA	Reference			NA	NA	NA
**CRP at BL [g/dl]**	0.29	0.05–0.76	0.06	0.4	0.06–1.04	0.21	0.42	0.10–0.89	0.12	0.49	0.1–1.11	0.26
**steroids at BL**				NA	NA	NA				NA	NA	NA
* yes	0.52	0.07–2.59	0.46	NA	NA	NA	1.56 * 10 ^-8^	NA	0.99	NA	NA	NA
* no	Reference			NA	NA	NA	Reference			NA	NA	NA
**VDZ level at W6 [µg/ml]**	1.06	1.0–1.13	0.04	1.05	0.98–1.13	0.21	1.06	1.0–1.13	0.04	1.06	0.99–1.16	0.13
The table summarizes the results of univariate and multivariate logistic regression which was used to investigate association between vedolizumab serum levels at week 6 and calprotectin remission at weeks 26 and 52. Variables that fulfilled pre-specified criteria for incorporation into multivariate modelling. NA –multivariate logistic regression was not performed, BL – baseline, CI – confidence interval, CRP – C-reactive protein, OR – odds ratio, W12 – week 12, W52 – week 52, VDZ – vedolizumab												

Due to the small number of patients both with increased serum CRP at baseline and CRP remission at week 12 and 52, associations with VDZ serum levels were not performed. Additionally, we did not observe associations between week 2 and week 6 VDZ serum levels and an aggregate endpoint of disease deterioration in the period between week 12 and 52, defined as “corticosteroid use OR new application of other immunosuppressive drugs OR VDZ treatment interval shortening apart from change from iv to sc formulation by patient’s request” (data not shown).

Previous studies showed that administration of VDZ 2mg/kg on days 1 and 29 was sufficient to reach almost complete saturation of α4β7 receptors 7 13 . In our cohort, no patient received a dose under 3 mg/kg at baseline (median 4.05 mg/kg, IQR = 1.99 mg/kg, data available for 45/58 patients) and thus baseline underexposure confounding our outcomes might be excluded.

Finally, the drug concentrations were compared between those patients who fulfilled any definition of an active disease at baseline (n = 53) with inactive ones (n = 5). At week 2 median VDZ levels for active patients were 22 µg/ml (IQR: 13.7 µg/ml, n = 38) and 40.9 µg/ml for inactive patients (IQR: 23.3 µg/ml, n = 5). At week 6 these values were 19.1 µg/ml (IQR: 12.7 µg/ml, n = 42) and 35.5 µg/ml (IQR 16.3 µg/ml, n = 5), respectively, and at week 12 15.9 µg/ml (IQR: 10.4 µg/ml, n = 29) for active patients as well as 11.8 µg/ml (IQR: 9.4 µg/ml, n = 4) for inactive ones. No statistically significant differences were observed. Thirteen patients who met all three disease activity criteria at baseline (“super-active cohort”) presented with nominally lower VDZ concentrations in the induction and early post-induction period in contrast to other 45 patients (statistically significant results only for week 6 data, p = 0.01, data not shown).

## Discussion

In this real-world evidence (RWE) study on CD patients treated with vedolizumab, we aimed at exploring associations between early drug exposure and therapeutic outcomes. Our results from a multivariate regression model did not provide evidence on a relationship between VDZ serum concentrations measured during the induction period and short- (week 12), mid- (week 26) or long-term (week 52) remission outcomes based on clinical activity or biomarker normalization. This finding is supported by lacking correlations between VDZ serum levels at week 2 and 6 and the need for corticosteroid use or dosing interval reductions of VDZ during the maintenance treatment.

Model-predicted VDZ exposure quartiles imputed from pivotal clinical trial data of GEMINI 2 and VISIBLE 2 described a flat, linear exposure-clinical remission profile for intravenous VDZ Q8W/Q4W maintenance dosing and a U-shape-like profile for subcutaneous Q2W maintenance dosing, respectively, with higher clinical remission rates at week 52 in the third versus the fourth trough level quartile (60.3% vs 50.7%) with subcutaneous administration 14 15 . A biologic underpinning of a biphasic exposure-response relationship of vedolizumab has been suggested. Becker et al 16 proposed that higher VDZ levels might impair regulatory T cells in a same way as effector T cells. This could lead to a reduced transmigration of regulatory T cells and decreased anti-inflammatory efficacy. However, this study was based on experiments with peripheral blood mononuclear cells ex vivo and the results were not validated in any in vivo model.

While exposure-clinical remission relationships were elaborated for induction (week 6) and post-maintenance (week 52) visits from the clinical trials data, real-world evidence (RWE) studies set out to establish early VDZ serum concentrations as predictor of VDZ maintenance treatment efficacy. In general, the former studies are building on observed PK parameters and outcome data from the same visit and, consequently, might be subjected to a selection bias towards patients with drug retention due to more favorable safety and/or efficacy. The latter by imputing also missing maintenance outcome data regardless of reason as non-response would pose a more conservative approach to evaluate exposure-response relationships. Our study falls into the latter category and is adding evidence to a string of discordant findings (see supplementary Table 1 for a list of studies exploring exposure-response relationships of VDZ in CD).

From the phase 4 prospective open-label multi-center LOVE CD trial, a pharmacokinetic-pharmacodynamic model of VDZ for targeting endoscopic remission was developed. In 74 mostly anti-TNF-α exposed patients, an individual predicted VDZ serum concentration of 20.0 mg/L at week 22 had a 35% probability of achieving endoscopic remission at week 26 17 . Although this is one of a few studies exploring an endoscopic outcome to VDZ treatment in CD, the visits for PK assessment and endoscopy were only 4 weeks apart, relativizing the clinical relevance of that finding. What could be the additional diagnostic yield to measure VDZ serum concentrations at week 20 if endoscopy is anyhow performed 4 weeks later, as any dose optimization attempts at week 20 would be unlikely to translate into incremental endoscopic improvement by week 24? A retrospective Belgian cohort study including 179 CD patients also suggested supporting evidence in early VDZ monitoring by reporting higher week 6 and week 14 VDZ serum concentrations in subjects who achieved mucosal healing, biologic remission or clinical remission 6 months post baseline in those with active disease 18 . However, the predictive value of the calculated VDZ serum level threshold was moderate with an area under the receiver operating characteristic (AUROC) < 0.7.

The results from our study are more in line with other RWE studies, in which no difference in VDZ serum levels during induction between remitters and non-remitters in the maintenance phase both for clinical and biochemical outcomes were observed 19 20 21 . Differences in outcome measures, timing of visits, previous exposures to biologics and concomitant small-molecule immunomodulators might contribute to the overall heterogeneity of findings from VDZ exposure-response relationship studies in CD 7 . Furthermore, various detection kits are used for the quantitation of VDZ serum concentrations which might impact study comparability 22 . Notably, VDZ levels in our patient cohort were generally lower than in other studies 23 . In addition, anti-vedolizumab antibodies were not measured in our study and in general data are scarce from other studies.

Sizeable RWE studies such as PANTS might delineate robust exposure-response relationships, though. In that study, loss of response at year 2 and 3 for patients treated with infliximab and adalimumab was predicted by low anti-TNF drug concentrations at week 14 in a multivariable model 24 . In contrast, the sufficient size is exactly an issue of RWE studies interrogating VDZ pharmacokinetic-outcome relationships in CD. This is particularly important if that relationship is supposed to be flat or U-shaped, drug serum concentrations are highly variable, and outcome parameters prone to subjectivity (PROs) and sample variability (fCP). Contingent upon those potential caveats, we do not categorically reject the hypothesis of a predictive value of early VDZ pharmacokinetics. In our study, patients with clinical remission at week 52 presented with numerically higher drug levels at weeks 2, 6 and 12. At least, we are ruling out VDZ under-exposure for target saturation in our patients as no patient received a dose under 3 mg/kg at baseline 7 . However, it is to note that a minimum drug exposure for α4β7 occupancy on peripheral and mucosal effector-memory T cells may be necessary but not sufficient for clinical efficacy of vedolizumab. 13 22 23 .

Finally, differences in baseline disease activity of recruited patients would influence the pharmacokinetics of VDZ and associations with outcome. An increased chronic inflammatory burden of the intestinal mucosal immune system results in intensified gut-associated lymphoid tissue priming of α4β7 lymphocytes which are recirculating to the inflamed gut 25 . Thus, we hypothesized that individuals with higher disease activity would have lower VDZ serum concentrations. In line with that reasoning, we observed lower VDZ serum concentrations in patients who presented with both clinical and biomarker disease activity. Of note, elevated fecal calprotectin was the predominant baseline disease activity criterion in our study.

The retrospective character of the study limits the interpretation of our results. PK samples with a longest interval of up to 2 days taken prior to drug administration qualified for our analysis, which might cause minor inconsistencies in the interpretation of exposure. As described above, sample size limits the robustness of our results. We included 58 individuals with some data missing, particularly from the mid- and late-maintenance period. On the other hand, our cohort included patients usually ineligible for clinical trials and often excluded also from real-world studies 26 , such as those without objective signs of inflammation but still requiring therapy after discontinuation of prior treatment due to intolerance or those with many previous, partially major surgeries. In line with the representativeness of a real-world tertiary referral site eligibility by clinical activity of our cohort was determined in the majority of the patients by a dominant PRO which was either abdominal pain OR elevated stool frequency. The clinical utility of this concept has been recently investigated in a post-hoc analysis and showed improved associations with a 1-year endoscopic remission 27 . Finally, we provided stringent definitions of remission and displayed VDZ serum concentration data from week 2 and week 6, time points that previously have rarely been measured and rarely reported in the context of CD, respectively.

In conclusion, we showed from our real-world cohort of Crohn’s disease patients that higher serum concentrations of vedolizumab measured in the induction period were not associated with significantly higher maintenance treatment efficacy. Nonetheless, the predictive value of early VDZ PK in CD needs to be further scrutinized in larger data sets utilizing objective end-point measures.

## Supplementary Material

Supplementary Material Zusatzmaterial
