# Supplementary material for: Post-induction serum vedolizumab levels are not associated with better maintenance outcomes in patients with Crohn’s disease
Source: Z Gastroenterol. 2026 Jan 12;64(2):157–67. doi: 10.1055/a-2744-5136 (PMC13077553; doi:10.1055/a-2744-5136)
Supplement: Supplementary file 1 — Supplementary Material [file 10-1055-a-2744-5136_27448953.pdf]

CITATION	CLINICAL TRIAL VS REAL-WORLD	PROSPECTIVE VS RETROSPECTIVE	NUMBER OF CD PATIENTS	OUTCOMES	CONCLUSIONS
**Sandborn WJ et al, NEJM 2013 (1)**	Clinical trial	Prospective	1115	CDAI response and remission W6 + VDZ level W6;<br>Clinical remission W52 + VDZ level W46	Numerically more patients in the fourth quartile who reached CDAI response; no differences in absolute numbers between the quartiles for other endpoints.
**Vermeire S et al, JCC 2022 (2)**	Clinical trial	Prospective	275	Clinical remission (CDAI score ≤150 at W52); enhanced clinical response defined as ≥100 decline in CDAI score from baseline at W52	Relationship between higher VDZ levels and proportion of patients in clinical remission W52 and enhanced clinical response W52.
**D'Haens G et al, Expert Rev Clin Pharmacol 2024 (3)**	Clinical trial	Prospective	1418	Clinical remission defined as CDAI ≤ 150 at W52	The rate of clinical remission in IV Q8W and IV Q4W dosing arms increased markedly with higher exposure, based on the quartile analysis. The clinical remission rate with SC Q2W treatment increased from 37.7% in Q1 to 63.2% with Q3 exposure but decreased to 49.3% with Q4 exposure.
**Vermeire S et al, JCC 2020 (4)**	Clinical trial	Prospective	88	Clinical remission defined as HBI ≤ 4 at W56	No differences in VDZ trough levels W56 between clinical remitters and non-remitters W56.
**Rosario M et al, JCC 2017 (5)**	Clinical trial	Prospective	1530	Clinical response and clinical remission at W6; clinical remission at W52	Median W6 VDZ levels slightly higher in remitters than in non-remitters at W6 in patients with CD from GEMINI 2 trial.<br><br>The exposure–efficacy relationship for clinical remission at W6 was shallower in patients with CD from

					GEMINI 3 than in patients from GEMINI 2. “Increasing vedolizumab trough concentrations from Q1 [≤ 17.1 µg/ml] to Q4 [> 32.5–128 µg/ml] resulted in an absolute increase in clinical remission rate of only approximately 5%.”
**Guidi L et a, UEG Journal 2019 (6)**	Real-world	Prospective	42	Clinical remission defined as HBI of ≤4 and without steroid treatment at W14, W22, W54; MH at W54.	No differences in W6 VDZ levels between W14, W22 and W54 clinical remitters and non-remitters; differences in W6 and W14 VDZ levels between MH at W54 and patients with no MH.
**Steenholdt C et al, J Gastroenterol and Hepatol 2024 (7)**	Real-world	Prospective	42	Steroid-free clinical response or remission at end of induction (W14) and steroid-free clinical, objective or combined clinical-objective remission at 1 year.	VDZ level comparison only for both IBD types, no discrimination between UC and CD.
**Hanzel J et al, UEG J 2019 (8)**	Real-world	Prospective	28	Combined endoscopic SES-CD < 4 without ulceration and clinical remission (resolution of abdominal pain), endoscopy performed between W20 and W54.	Differences in VDZ levels at W22 between remitters and non-remitters; no differences in W2, W6, W14, W38 and W54 VDZ levels.
**Ungaro R C et al, JCC 2019 (9)**	Real-world	Prospective	142	Corticosteroid-free clinical and biochemical remission defined as a composite of clinical remission, a normalized CRP and no oral corticosteroid use in the previous 4 weeks. Clinical remission as a HBI score of ≤ 4. Deep remission as corticosteroid-free clinical remission with normal CRP and endoscopic remission.	No difference in VDZ levels between remitters and non-remitters (for corticosteroid-free remission and CRP normalization).<br><br>Higher VDZ levels for those who reached corticosteroid-free endoscopic remission compared to non-remitters.

					Higher VDZ levels for those who reached corticosteroid-free deep remission compared to non-remitters.
**Al-Bawardy B et al, JCC 2019 (10)**	Real-world	Retrospective	106	Clinical remission based on the "chart reviews of the treating physician's overall assessment". Deep remission defined as combined clinical remission and MH; VDZ levels median of 38.5 days after the last dose - efficacy assessment at the same time as VDZ measurement	Higher VDZ levels for those who reached CRP normalization compared to those who did not. No difference for clinical remission and MH.
**Ungar B et al, CGH 2018 (11)**	Real-world	Prospective	67	Clinical remission defined as HBI ≤ 4. Clinical response - decrease of ≥3 points in HBI. Primary nonresponse defined as cessation of vedolizumab therapy by W14 owing to a lack of clinical response.	Higher W6 VDZ levels for those who reached clinical remission at W6 compared to non-remitters.<br><br>No association between W2 and W6 VDZ levels and clinical remission by the end of induction.
**Sivridas M et al, Pharmaceutics 2023 (12)**	Real-world	Prospective	59	Clinical remission defined as HBI ≤ 4, biochemical remission as CRP < 5 mg/L and fecal calprotectin < 250 mg/kg – „maintenance efficacy".	No differences in VDZ levels between clinical remitters and non-remitters, also no difference for biochemical remission.
**Hüttemann E et al, J Clin Med 2024 (13)**	Real-world	Retrospective	23	Clinical response defined as a substantial improvement in disease symptoms; clinical remission - complete absence of symptoms. "To assess the clinical outcomes, the treating physician took into account the patient's symptom burden, biomarkers, and imaging; no score was used. Endoscopic response and	No difference in W6 VDZ levels between clinical responders and non-responders after 6 and 12 months.<br><br>No differences in W6 VDZ levels between endoscopic responders and non-responders after 6 and 12 months.

				remission were verified through endoscopy findings during follow-up and were supported by histopathologic findings when appropriate".	No discrimination between UC and CD patients.
**Vaughn B P et al, J Clin Med 2020 (14)**	Real-world	Retrospective	87	Clinical response after at least one infusion at the escalated dose, as assessed by the treating physician.	Association between VDZ levels and clinical response at the time of the measurement.<br><br>No discrimination between UC and CD patients.
**Dreesen E et al, CGH 2018 (15)**	Real-world	Retrospective	113	Endoscopic healing (absence of ulcers), clinical response (physician global assessment); biologic remission defined as CRP ≤ 5 mg/L at W22.	Higher W6 VDZ levels between biological remitters compared to non-remitters at W6.<br><br>Higher W10 VDZ levels in biological remitters compared to non-remitters at W6.<br><br>Higher W14 VDZ levels in biological remitters compared to non-remitters at W14.<br><br>Higher W22 VDZ levels in endoscopic remitters compared to non-remitters at W22.<br><br>Higher W22 VDZ levels in biological remitters compared to non-remitters at W22.
**Hanzel J et al, Inflamm Bowel Dis 2022 (16)**	Clinical trial	Prospective	108	SES-CD at W26 at W52.	Higher W22 VDZ levels associated with W26 endoscopic remission.

**Vande Casteele N et al, Aliment Pharmacol Treatment 2022 (17)**	Real-world	Retrospective	391	Clinical remission defined as PGA; endoscopic remission defined as the absence of large ulcers >5 mm; deep remission - clinical + endoscopic remission at W14, W26 and W52.	Higher W14 VDZ levels in clinical remitters at W14 compared to non-remitters.<br><br>Higher W46 VDZ levels in clinical remitters at W52 compared to non-remitters.<br><br>Higher W6 VDZ levels in endoscopic remitters at W14 compared to non-remitters.<br><br>Higher W10 VDZ levels in endoscopic remitters at W14 compared to non-remitters.<br><br>Higher W14 VDZ levels in endoscopic remitters at W14 compared to non-remitters.
**Yarur A J et al, Dig Dis Sci 2019 (18)**	Real-world	Prospective	25	Steroid-free endoscopic remission (SES-CD ≤ 2) at W52.	Higher W2 and W6 VDZ levels in remitters at W52 compared to non-remitters.<br><br>No discrimination between UC and CD patients.
**Verstockt B et al, JCC 2020 (19)**	Real-world	Retrospective	179	Endoscopic remission at (absence of ulcerations) W22 endoscopic remission; biological remission defined as as a CRP ≤5 mg/L.	Higher W6 VDZ levels in biological remitters at W22 compared to non-remitters.<br><br>Higher W6 VDZ levels in endoscopic remitters W22 compared to non-remitters.

					Higher W14 VDZ levels in biological remitters at W22 compared to non-remitters.<br><br>Higher W14 VDZ levels in endoscopic remitter at W22 compared to non-remitters.

Supplementary Table 1. Summary of all available publications on association between vedolizumab serum concentration and therapeutic outcomes in patients with Crohn's disease. CD – Crohn's disease, CDAI – Crohn's disease activity index, CRP – C-reactive protein, HBI – Harvey-Bradshaw Index, IQ – intravenous, MH – mucosal healing, PGA – Physician Global Assessment, Q1 – first quartile, Q2 – second quartile, Q3 – third quartile, Q4 – fourth quartile, Q2W – every 2 weeks, Q4W – every 4 weeks, Q8W – every 8 weeks, SC – subcutaneous, SES-CD – Simple Endoscopic Score for Crohn's Disease, UC – ulcerative colitis, VDZ – vedolizumab, W - week

REFERENCES

1. Sandborn WJ, Feagan BG, Rutgeerts P, Hanauer S, Colombel JF, Sands BE, et al. Vedolizumab as induction and maintenance therapy for Crohn's disease. N Engl J Med. 2013;369(8):711-21.

2. Vermeire S, D'Haens G, Baert F, Danese S, Kobayashi T, Loftus EV, et al. Efficacy and Safety of Subcutaneous Vedolizumab in Patients With Moderately to Severely Active Crohn's Disease: Results From the VISIBLE 2 Randomised Trial. J Crohns Colitis. 2022;16(1):27-38.

3. D'Haens G, Rosario M, Polhamus D, Dirks NL, Chen C, Kisfalvi K, et al. Exposure-efficacy relationship of vedolizumab subcutaneous and intravenous formulations in Crohn's disease and ulcerative colitis. Expert Rev Clin Pharmacol. 2024;17(4):403-12.

4. Vermeire S, Lukáš M, Magro F, Adsul S, Lindner D, Rosario M, et al. Vedolizumab Efficacy, Safety, and Pharmacokinetics With Reduced Frequency of Dosing From Every 4 Weeks to Every 8 Weeks in Patients With Crohn's Disease or Ulcerative Colitis. J Crohns Colitis. 2020;14(8):1066-73.

5. Rosario M, French JL, Dirks NL, Sankoh S, Parikh A, Yang H, et al. Exposure-efficacy Relationships for Vedolizumab Induction Therapy in Patients with Ulcerative Colitis or Crohn's Disease. J Crohns Colitis. 2017;11(8):921-9.

6. Guidi L, Pugliese D, Panici Tonucci T, Bertani L, Costa F, Privitera G, et al. Early vedolizumab trough levels predict treatment persistence over the first year in inflammatory bowel disease. United European Gastroenterol J. 2019;7(9):1189-97.

7. Steenholdt C, Lorentsen RD, Petersen PN, Widigson ES, Kloft C, Klaasen RA, Brynskov J. Therapeutic drug monitoring of vedolizumab therapy in inflammatory bowel disease. J Gastroenterol Hepatol. 2024;39(6):1088-98.

8. Hanžel J, Sever N, Ferkolj I, Štabuc B, Smrekar N, Kurent T, et al. Early vedolizumab trough levels predict combined endoscopic and clinical remission in inflammatory bowel disease. United European Gastroenterol J. 2019;7(6):741-9.

9. Ungaro RC, Yarur A, Jossen J, Phan BL, Chefitz E, Sehgal P, et al. Higher Trough Vedolizumab Concentrations During Maintenance Therapy are Associated With Corticosteroid-Free Remission in Inflammatory Bowel Disease. J Crohns Colitis. 2019;13(8):963-9.

10. Al-Bawardy B, Ramos GP, Willrich MAV, Jenkins SM, Park SH, Aniwan S, et al. Vedolizumab Drug Level Correlation With Clinical Remission, Biomarker Normalization, and Mucosal Healing in Inflammatory Bowel Disease. Inflamm Bowel Dis. 2019;25(3):580-6.

11. Ungar B, Kopylov U, Yavzori M, Fudim E, Picard O, Lahat A, et al. Association of Vedolizumab Level, Anti-Drug Antibodies, and α4β7 Occupancy With Response in Patients With Inflammatory Bowel Diseases. Clin Gastroenterol Hepatol. 2018;16(5):697-705.e7.

12. Sivridaş M, Creemers RH, Wong DR, Boekema PJ, Römkens TEH, Gilissen LPL, et al. Therapeutic Drug Monitoring of Vedolizumab in Inflammatory Bowel Disease Patients during Maintenance Treatment-TUMMY Study. Pharmaceutics. 2023;15(3).

13. Hüttemann E, Muzalyova A, Gröhl K, Nagl S, Fleischmann C, Ebigbo A, et al. Efficacy and Safety of Vedolizumab in Patients with Inflammatory Bowel Disease in Association with Vedolizumab Drug Levels. J Clin Med. 2023;13(1).

14. Vaughn BP, Yarur AJ, Graziano E, Campbell JP, Bhattacharya A, Lee JY, et al. Vedolizumab Serum Trough Concentrations and Response to Dose Escalation in Inflammatory Bowel Disease. J Clin Med. 2020;9(10).

15. Dreesen E, Verstockt B, Bian S, de Bruyn M, Compernolle G, Tops S, et al. Evidence to Support Monitoring of Vedolizumab Trough Concentrations in Patients With Inflammatory Bowel Diseases. Clin Gastroenterol Hepatol. 2018;16(12):1937-46.e8.

16. Hanzel J, Dreesen E, Vermeire S, Löwenberg M, Hoentjen F, Bossuyt P, et al. Pharmacokinetic-Pharmacodynamic Model of Vedolizumab for Targeting Endoscopic Remission in Patients With Crohn Disease: Posthoc Analysis of the LOVE-CD Study. Inflamm Bowel Dis. 2022;28(5):689-99.

17. Vande Casteele N, Sandborn WJ, Feagan BG, Vermeire S, Dulai PS, Yarur A, et al. Real-world multicentre observational study including population pharmacokinetic modelling to evaluate the exposure-response relationship of vedolizumab in inflammatory bowel disease: ERELATE Study. Aliment Pharmacol Ther. 2022;56(3):463-76.

18. Yarur AJ, Bruss A, Naik S, Beniwal-Patel P, Fox C, Jain A, et al. Vedolizumab Concentrations Are Associated with Long-Term Endoscopic Remission in Patients with Inflammatory Bowel Diseases. Dig Dis Sci. 2019;64(6):1651-9.

19. Verstockt B, Mertens E, Dreesen E, Outtier A, Noman M, Tops S, et al. Influence of Drug Exposure on Vedolizumab-Induced Endoscopic Remission in Anti-Tumour Necrosis Factor [TNF] Naïve and Anti-TNF Exposed IBD Patients. J Crohns Colitis. 2020;14(3):332-41.
